# Application of Artificial Intelligence to the Prediction of the Antimicrobial Activity of Essential Oils

**DOI:** 10.1155/2015/561024

**Published:** 2015-09-17

**Authors:** Mathieu Daynac, Alvaro Cortes-Cabrera, Jose M. Prieto

**Affiliations:** Center for Pharmacognosy and Phytotherapy, Department of Pharmaceutical and Biological Chemistry, School of Pharmacy, University of London, 29-39 Brunswick Square, London WC1N 1AX, UK

## Abstract

Essential oils (EOs) are vastly used as natural antibiotics in Complementary and Alternative Medicine (CAM). Their intrinsic chemical variability and synergisms/antagonisms between its components make difficult to ensure consistent effects through different batches. Our aim is to evaluate the use of artificial neural networks (ANNs) for the prediction of their antimicrobial activity.* Methods.* The chemical composition and antimicrobial activity of 49 EOs, extracts, and/or fractions was extracted from NCCLS compliant works. The fast artificial neural networks (FANN) software was used and the output data reflected the antimicrobial activity of these EOs against four common pathogens:* Staphylococcus aureus, Escherichia coli, Candida albicans*, and* Clostridium perfringens* as measured by standardised disk diffusion assays.* Results.* ANNs were able to predict >70% of the antimicrobial activities within a 10 mm maximum error range. Similarly, ANNs were able to predict 2 or 3 different bioactivities at the same time. The accuracy of the prediction was only limited by the inherent errors of the popular antimicrobial disk susceptibility test and the nature of the pathogens.* Conclusions.* ANNs can be reliable, fast, and cheap tools for the prediction of the antimicrobial activity of EOs thus improving their use in CAM.

## 1. Introduction

Essential oils (EOs) are endowed with potent, antioxidant, antimicrobial, and anti-inflammatory properties [1, 2], making them convenient “3-in-1” active ingredients in a plethora of Complementary and Alternative Medicine preparations. These properties underpin active research and development resulting in an ever-increasing number of works reporting on both their composition and bioactivity. However research on essential oils suffers from their inherent intraspecific variability composition which depends—among other factors—on the location, altitude, meteorology, and type of soil, thus resulting in a high rate of irreproducible reports. Furthermore, the evaluation of the bioactivities of EOs cannot be always attributed to one single compound in the mixture. Synergisms and antagonisms have been consistently reported between the constituents of EOs [3, 4].

In fact, the prediction of the bioactivity of EOs after their unique chemical composition is an idea already well established among the scientific community [3] but not systematically explored yet, due to the experimental complexity of characterising all possible chemical interactions between dozens of EOs components and the microbes.

A computational tool allowing for the selection of EOs with similar antimicrobial effects, regardless of their chemical variations and without the need of laboratory analysis, would result in savings and an enhanced consistency of the final product. This tool should also be able to take into account possible (bio)chemical interactions, synergisms, and antagonisms between the oil's components and the microbes. The use of computational models such as artificial neural networks (ANNs) holds potential to overcome all these challenges. In fact, we already demonstrated that the prediction of the antioxidant properties of EOs in two experimental biochemical models is possible [5].

ANNs are composed by a set of computer-generated virtual/artificial neurons organized in interconnected layers. Each neuron has a specific weight in the processing of the information. The optimal weights are calculated with available pairs of input and output data constituting the training set. Using these pairs, the ANN is able to minimize output error modifying weights as required. While two of these layers are connected to the “outside world” (input layer, where data is presented and output layer, where a prediction value is obtained), the rest (hidden layers) are defined by neurons connected to each other, avoiding connections between neurons of the same layer. In our case the inputs are the different proportions of each chemical in the EO whilst the output is the inhibition zone provided by the whole EO.

To our knowledge, the use of ANNs in microbiology has been quite restricted to the modelling of microorganism growth [6–8]. In this work we aim at using this computational approach to predict the antimicrobial properties of complex chemical mixtures on a panel of microorganisms.

## 2. Materials and Methods

### 2.1. Data Retrieval

We selected scientific reports using the National Committee for Clinical Laboratory Standards [9] standardized method for zone diameter measurements reporting similar inhibitory values for the same antibiotics of reference.

A total of 18 articles [10–27] meeting these criteria were found, providing 49 couples of inputs (composition) and outputs (antimicrobial activity) data against one or more of 14 pathogens (see Table S1 in Supplementary Material available online at http://dx.doi.org/10.1155/2015/561024) of which we only selected* Staphylococcus aureus* ATCC 25923 (facultative anaerobic Gram+ cocci),* Escherichia coli* ATCC 25922 (facultative anaerobic Gram− bacillus),* Clostridium perfringens* Kukens Turkey (anaerobic Gram+ bacillus) (abbreviated in the tables as* S. perfringens* KT), and* Candida albicans* ATCC 10239 (yeast).

### 2.2. Selection of Inputs

**Figure 1:**
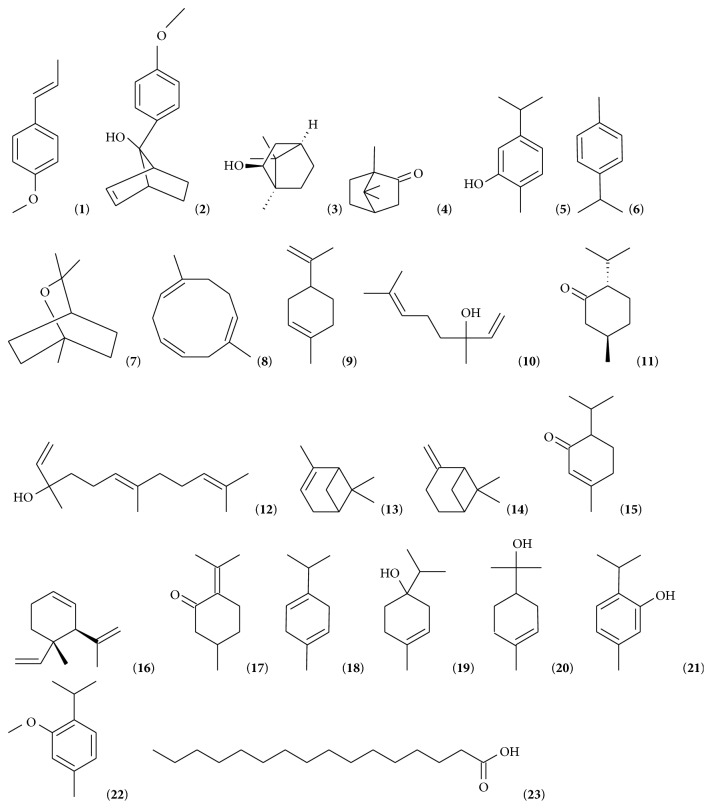
Chemical structures of the chemicals with proven antimicrobial activity present in selected EOs and plant extracts.

The chemical composition of the selected EOs included up to 179 different compounds. In order to avoid excessive complexity of the neural network and minimize the associated structural problems, it was necessary to reduce the number of inputs. Two considerations were made: either to retain the compounds with known antimicrobial properties only (small data set, Figure 1) or to eliminate the compounds without known antimicrobial activity and/or present at very low percentages (≤ 5%) (larger input data set, Table S2, Supplementary Data).

Using the first strategy, only 22 volatile compounds with proven antimicrobial activity were retained (Figure 1): (*E*)-anethole (**1**), syn-7-hydroxy-7-anisylnorbornene (**2**), borneol (**3**), camphor (**4**), carvacrol (**5**), p-cymene (**6**), eucalyptol (**7**), geijerene (**8**), limonene (**9**), Linalool (**10**), menthone (**11**), nerolidol or peruviol (**12**), *α*-pinene (**13**), *β*-pinene (**14**), piperitone (**15**), pregeijerene (**16**), pulegone (**17**),* g*-terpinene (**18**), terpinen-4-ol (**19**), *α*-terpineol (**20**), thymol (**21**), and thymol methyl ether (**22**). In addition we included palmitic acid (**23**). This is not an EOs component, but its presence is reported in some plant extracts together with volatiles components. Its antimicrobial properties against a wide arrays of microbes have been recently reviewed [28].

On the other hand, by eliminating the minor compounds without known/reported antimicrobial activity, we brought the input components down to 75 for* S. aureus*, 78 for* E. coli*, 47 for* C. albicans,* and 51 for* C. perfringens*. This option assumes that their contribution to the global antimicrobial activity and potential synergistic/antagonistic effects could be neglected* a priori*, just because they are in too low quantity. In this way, the 23 principal antimicrobial agents forming the previous set are still present in this one, escorted by other major compounds which characterize the EOs. The minimum concentration was set up to 5% of the EO composition (see Table S2 in Supplementary Data for a list of the retained chemicals).

### 2.3. Design of the ANN

ANNs were developed and run on a personal computer using fast artificial neural network software ver. 1.2.0-1. This package was downloaded from its original repository (http://leenissen.dk/fann/) and installed following the guidelines provided by the developers in a Dell OptiPlex GX620 under Ubuntu 7.04 and Microsoft Windows XP Professional SP2.

FANN is a free, open source neural network library, which implements multilayer artificial neural networks in C language with support for both fully connected and sparsely connected networks. Cross-platform execution in both fixed and floating point is supported [29]. We chose this software because it is versatile, well documented, and fast as well as including a framework for easy handling of training data sets.

Several different designs of feed forward, multilayer, and back-propagation ANNs with a variable number of input neurons depending on different choice criteria of the EOs components, a variable number and topology of hidden layers, and one or more neurons in the output layer were tested (see Figure S3, Supplementary Data). The internal parameters were as described in our previous paper [5]: activation functions were set to FANN_SIGMOID_SYMETRIC for both hidden and output neurons, and training was based on a supervised method with back-propagation strategy, learning rate 0.7, minimum error 0.0001, and maximum epochs to 500000.

The data were transformed into a suitable interval (0 to 1) for input and output neurons by dividing their value by 100. The training-validating tests were run in automated manner by using an in-house program in C language developed by one of the authors (Cortes-Cabrera). The most accurate networks were selected as potential candidates and further validated.

We used the following criteria in the interpretation of the accuracy of the predictions, based on their deviations from the real inhibitory diameters (ΔID): ΔID ≤ 5 mm was considered as very accurate, ΔID ≤ 10 mm represent accurate predictions, and ΔID > 10 mm would mean mistaken predictions.

In addition, we compared the results of ANNs designed with FANN with the results obtained using a commercial software. For this purpose, NeuralWorks Predict was chosen. This is an integrated, state-of-the-art tool for rapidly creating and deploying prediction and classification applications. It incorporates years of modelling and analysis experience gained from working with a wide variety of analysis and interpretation problems. Conveniently, this software is fully integrated within the Microsoft Office environment, running as an Add-in of Microsoft Excel. NeuralWorks Predict claims to combine neural network technology with genetic algorithms, statistics, and fuzzy logic to automatically find optimal or near-optimal solutions for a wide range of problems [29]. An evaluation copy of NeuralWorks Predict (PredictDemo) was downloaded from its official repository (http://www.neuralware.com/). PredictDemo has the same functionality of NeuralWorks Predict although limited to a maximum of 32 input neurons. Therefore we used this tool with the small input data set (*n* = 23) shown in Figure 1. All the available parameters for the type of input data namely “noise,” “data transformation,” and “network search” were tested and the best options kept for further work.

## 3. Results

### 3.1. Optimisation of the ANN Design and Input Data Set in FANN

In PredictDemo the ANN is internally designed by the software as to better predict the data and its topology is not accessible to the user. Contrarily, in FANN the user has to design the topology, so a preliminary study of the performance of different network topologies had to be done. For this purpose when using the small input data set of 23 components we kept the same topology for the hidden layers, namely, 20→15→7, as described in our previous work [5].

When using the large input data set, we preliminary tested different topologies with 51 input components predicting the antimicrobial activity against* C. perfringens*. The network with the most accurate predictions for each topology has been chosen among ten, and the average of three different predictions led to the following results: 30→15→5 (43% accuracy), 40→30→20→10 (28% accuracy), 20→30→5 (18% accuracy), 20→20→20 (7% accuracy), and 10→20→30 (4% accuracy). Therefore the 30→15→5 topology was kept for the rest of the experiments with this input data set.

### 3.2. Prediction of the Antimicrobial Activities of Essential Oils (1 Output)

The ability of open source and commercial ANNs to predict the antimicrobial activity of very complex natural products on selected microorganisms was compared using the same inputs (small input data set of 23 components) and the same learning/validating sets: 30/6 for* S. aureus*; 32/7 for* E. coli*; 31/6 for* C. perfringens*; and 30/5 for* C. albicans*. The overall accuracy of the predictions is shown in Table 1 (PredictDemo) and Table 2 (FANN). The Linear Regression Analysis for all the experiments using PredictDemo is presented in Figures and Tables S4–S8 (Supplementary Data) whilst those corresponding to experiments using FANN are presented in Figures and Tables S9–S11 (Supplementary Data).

The influence of the different input data set on the accuracy of the predictions can be discussed by comparing Table 2 (FANN, small data set) and Table 5 (FANN, large data set).

Three different experiments per microorganism, each with different learning sets, were run in both types of software. Conditions for FANN are described in Material and Methods and only the best of 10 different networks was chosen for each test. For PredictDemo the most accurate predictions have been observed with this combination of parameters: very noisy data, comprehensive data transformation, exhaustive variable selection, and exhaustive network search.

During the training, PredictDemo was able to create artificial neural networks from the input data and to establish a reliable correlation between the inputs and the outputs: 10 of the 12 ANNs created present a regression coefficient *R* ^2^ > 0.90 (Figure S2, Supplementary information). Also the inhibition diameters predicted by the software for all selected microorganisms are not incongruous; that is, the predicted inhibitory diameters stay in the same range of values than the real ones, namely, between 0 and 60 mm (Table S3, Supplementary information). Although the correlation obtained in the validating set was lower than for the learning set (Figure S4, Supplementary information), the prediction for the validating sets was “very accurate” (ΔID < 5 mm) in 36 out of 72 cases and “accurate” (ΔID < 10 mm) for more than two-thirds. However, nearly one-third of the predictions done by this software were far from the real values (ΔID > 10 mm). Overall, PredictDemo was more able to predict the inhibition diameters for* S. aureus* and* C. perfringens* than for* E. coli* and* C. albicans*.

The first learning set, consisting of EOs on which the 23 compounds with known antimicrobial activity represented the higher percentage within the oil, allowed the ANN to better predict antimicrobial activities, even if the validating set consisted of EOs in which the same components accounted for a low percentage of the total composition.

Overall, the predictions made by the two different computational approaches were similar, with nearly half of the predictions less than 5 mm away from the real inhibition disk diameters; exactly the same percentage (70%) could be considered as accurate (ΔID < 10 mm) and nearly one-third inaccurate (ΔID > 10 mm). A closer look to their performance per microorganism reveals that the prediction of antimicrobial properties of EOs against* S. aureus* is the most accurate regardless of the software, closely followed by predictions against* C. perfringens*. However there is a higher degree of error in the predictions of the antimicrobial properties against* E. coli* and* C. albicans*. Therefore, in order to analyse the influence of the different choices of learning sets in the training and performance of the ANNs, we compared their predictions against* S. aureus*. This shows that PredictDemo performed better when trained with the set of oils containing the highest percentage of known antimicrobial compounds, whilst FANN performed better if a random choice of EOs was used for the training (Tables 3 and 4).

Predictions were now attempted with the large input data set in FANN. This required a change of the topology of the ANN as to accommodate chemicals between 47 and 78 in the input layer. In this case, 3 different learning and validating sets were created randomly for each microorganism and ANN.

Overall the predictions of the inhibition disk diameter were more accurate with this new compound selection than in the previous experiments with both PredictDemo and FANN using the small input data set (Table 5). It can be seen that the larger input data set allowed for nearly two-thirds of the predictions to be less than 5 mm away from the real values, whilst more than 80% were in a range of ΔID < 10 mm. Almost all the predictions could be considered in agreement with the antimicrobial activities reported by the selected literature (93% with ΔID < 15 mm).

These results may indicate that this choice of chemicals is more representative in terms of the antimicrobial activity of the EOs. An interesting outcome is also the higher accuracy for* E. coli* (81%  ΔID ≤ 5 mm) when compared with the previous experiments (47.6% with FANN and 33.3% with PredictDemo). Predictions against* C. albicans* are also very much improved, but the difficulty in modelling the sensitivity of this microorganism within 5 mm of error is still evident.

### 3.3. Simultaneous Prediction of the Antimicrobial Activities of Essential Oils in Two or More Microorganisms (2 or 3 Outputs)

ANNs with 2 or 3 outputs were used to test the ability of the FANN software to predict antimicrobial activity of two or more pathogens at the same time, respectively. Consequently, the learning and validating sets of data came from articles in which the antimicrobial activity had been tested on the 2 or 3 pathogens of interest. The availability of such papers determined the choice of* S. aureus* and* E. coli* as outputs for the first test and* S. aureus*,* E. coli,* and* C. perfringens* as outputs for the second one. The number of available data allowed for 3 different learning and validating sets randomly selected, giving 17 “two-outputs” experiments, and 12 “three-outputs” experiments. The L/V sets followed the same 5 : 1 split used in the “one output” experiments.

The results show that two outputs (= simultaneous predictions) did not significantly limit the prediction ability of the artificial neural network (see Table 6 for summary and Figures and Tables S12-S13 in Supplementary Data for all results). Overall, the predictions were even more accurate than those of ANNs with one output only: predictions of the antimicrobial activity on* S. aureus* were again very accurate. For* E. coli*, a less significant correlation was obtained. However, still 58.8% of the predictions were accurate (ΔID ≤ 5 mm) and none more than 10 mm far from the real value. Here we need to take into consideration that the learning and validating sets were different, so we cannot draw any conclusion about the FANN software being more or less able to predict antimicrobial activity with 1 or 2 outputs at the same time. Whether these particularly accurate predictions observed in these experiments could be due to a more reliable data selection, which may limit the variability due to the common sources or error in calculating the antimicrobial activity, needs to be ascertained as soon as a larger number of available data in literature is made available.

In Table 7 we show clearly that FANN is still able to make accurate predictions of antimicrobial activity against three pathogens at the same time. The most accurate predictions are still observed for* S. aureus* whilst the prediction accuracy for the other pathogens was in the average of the previous predictions.

## 4. Discussion

### 4.1. Suitability of ANNs for the Prediction of Antimicrobial Activities

EOs act upon microorganisms through a not yet well defined mixture of both specific and unspecific mechanisms. In this regard, ANNs are a very good option as they have been successfully applied to processes with complex or poorly characterised mechanisms, as they only take into account the causing agent and its final effect [8, 30].

Indeed, the antibiotic activities of EOs depend on a complex chemistry and a poorly characterised mechanism of action. Different monoterpenes penetrate through cell wall and cell membrane structures at different rates, ultimately disrupting the permeability barrier of cell membrane structures and compromising the chemiosmotic control [31]. It is therefore conceivable that differences in the gram staining would be related to the relative sensitivity of microorganism to EOs. However, this generalisation is controversial as illustrated by conflicting reports in the literature. Nakatani [32] found that Gram-positive bacteria were more sensitive to EOs than Gram-negative bacteria, whereas Deans and Ritchie [33] could not find any differences related to the reaction. The permeability of the membrane is only one factor and the same EO may act by different mechanisms upon different microorganisms. As an example, the EO of* Melaleuca alternifolia* (tea tree), which inhibited respiration and increased the permeability of bacterial cytoplasmic and yeast plasma membranes, also caused potassium ion leakage in the case of* E. coli* and* S. aureus* [34].

In addition, ANNs are theoretically able to take into account synergies and antagonisms between inputs. There is a consistent body of data on many crude essential oils being more active than their separated fractions or components, reported on synergies. In some cases synergistic activity between two or three components could be experimentally demonstrated [35, 36], but to do so with dozens of chemicals is beyond reach.

Our results reflect the variability in the susceptibility of different microorganisms to the same EOs, but more importantly point towards some general trends. The antimicrobial effects of EOs upon* S. aureus* and* C. perfringens* (Gram+) were accurately modelled by our ANNs, thus meaning a clear relationship between the chemistry of EOs and their susceptibility, perhaps suggesting a more additive, physical, rather than pharmacological, mechanism of action. This also opens the prospect for further studies in order to ascertain the best set of volatile components providing optimum antimicrobial activity against these two pathogens and/or Gram+ in general. On the other hand, the lower accuracy of the predictions against* E. coli* (Gram−) and* C. albicans* (yeast) may suggest more complex pharmacological actions of the chemicals. In this case the activity may be pinned down to one or few active principles acting individually or in synergies.

### 4.2. Internal Factors Influencing the Accuracy of the Predictions

The challenge in modelling the activity of essential oils is mainly the selection of inputs and the topology. Ideally the data set would necessarily include all components of all essential oils. This adds a tremendous complexity to the network and, in fact, the number of inputs used in other ANN models is classically far lower than the set we had to deal with. On the other hand, the restriction of the input data set inevitability leads to a bias, but it is the only way forward in order to overcome this problem. Also the restricted number of comparable data present in the literature results in a low number of learning and validating sets. These factors do not invalidate the use of ANNs but limit any generalisation of the results [8]. Then again, we here aim at exploring the potential of ANNs to predict complex biological activities and the strategies to select the input data only as well as encouraging other researchers to apply ANNs to the field of CAM and microbiology in general. Achieving a critical mass of L/V sets to overcome these shortfalls would only be possible with the collaboration of a network of partners working towards creating an ANN dedicated to a particular product/task. Considering this, our contribution to the future use of ANNs in this area is that (1) selection of the inputs should consider all chemicals present above the threshold value of 5% of the total EO regardless if they are reported to be endowed with antimicrobial properties, and (2) feed forward back propagation ANNs using SIGMOID functions and one or more outputs are valid starting points for future works.

### 4.3. External Factors Influencing the Accuracy of the Predictions

In quantitative terms, the overall performance of the ANNs in this work (Antimicrobial activity of EOs) is lower than the one achieved in our previous work [5] (IC_50_s in biochemical assays). This could be explained by the following factors: (a) the highest degree of variability in the response of whole living organisms versus the higher reproducibility of biochemical reactions, (b) the intrinsic variability of the disk diffusion assays, (c) the difficulty in finding comparable data in the literature, and (d) the physicochemical incompatibility of EOs and microbiological media.

As discussed above, the variability of the biological response is mirrored by the fact that ANNs achieve better performance in predicting antimicrobial activities against microbes than yeasts. However, this may be also linked to the existence of more abusive reports (ΔID > 60 mm) for* C. albicans* which contributed to a higher error in the outputs. It is however true that the disk diffusion assay seems to be particularly suitable for* S. aureus*, as they show a higher interlaboratory reproducibility of the inhibitory areas reported for this microorganism [37].

Regarding the variability in the results, the National Committee for Clinical Laboratory Standards [9] recognises that an important number of factors may induce errors in the popular disk diffusion assays. These factors include contamination or other changes in the control strain,* inoculum* suspensions that are too heavy or too light, incorrect incubation temperature or atmosphere, loss of disk potency during handling or storage in the laboratory, and even clerical error in transcribing the quality control data/reader error in measuring zone diameters. Our first aim was to model the Minimum Inhibitory Concentration test rather than the inhibition zones one. Surprisingly, there are a very limited number of them. To complicate matters further, there is an apparent lack of agreement in the standard antibiotic of choice, resulting in yet fewer comparable papers. These technical limitations together with a worrying lack of consensus in terms of adherence to reference antibiotics renders much of the literature data on natural products research invalid for meta-analysis. In any case the volatility and poor solubility of most essential oils are problematic in methods that rely on diffusion or dilution of the test substance in a microbiological medium.

### 4.4. Concluding Remarks

The variability in composition and activity inherent to crude essential oils affect their application as antimicrobials. Delaquis and coworkers proposed to increase their reliability by adjusting the levels of their components to provide the required strength and spectrum of inhibition. These authors experimented with mixing individual fractions to consistently achieve a desired level of activity [38]. However, this strategy implies adding previous steps—and cost—to their manufacture and eventually do not avoid the need of laboratory analysis to ascertain their actual antimicrobial activity. We report here on an opposite strategy: using AI tools the manufacturer would be able to choose among all crude EOs offered in the market and the one providing the same functional activity of previous batches, regardless of their seasonal or geographical variation in composition, by predicting in seconds their effectiveness against a range of microorganisms.

Our results demonstrate the potential of ANNs as a tool to accomplish this aim and suggest strategies on the selection of inputs and conditions for the* in silico* experiments. They also gain insight into the limitations of the scientific data so far available—suffering from little standardization of the conditions in terms of reference antibiotics—as well as the shortfalls of the disk diffusion assay as an analytical tool.

Commercial AI tools may offer convenience and performance if properly trained and validated. They have a user friendly interface, which may be their only asset because in our hands similar free, open access tools are able to perform at the same level of accuracy. Perhaps this accuracy may be improved by ANNs dedicated to a reduced number of essential oils from a phylogenetically related group of plants only. Artificial intelligence holds promise as fast, cheap, and reliable tools to model the functional properties of complex natural products such as EOs, ensuring their activity batch after batch despite differences in composition thus resulting in a better evidence-based medical use.

## Supplementary Material

Supplementary Data file contains the composition of the inputs (chemicals), the validation of the ANNs (linear regresions) and the correaltion between real values and predictions. The Excel file contains the raw data with the full composition of the essential oils.

## Figures and Tables

**Table 1:** Prediction by PredictDemo of the inhibition diameter in disk diffusion assays for a specific microorganism (1 output) using the small data input set (*n* = 23). Accuracy is expressed in the percentage of predictions within the given error margin. In parenthesis, total of validating sets correctly predicted within the corresponding error margin.

Strains	Accuracy %			
	ΔID ≤ 5 mm	ΔID ≤ 10 mm	ΔID ≤ 15 mm	ΔID > 15 mm
*S. aureus* ATCC 25923	72.2 (13)	88.9 (16)	94.4 (17)	5.6 (1)
*E. coli* ATCC 25922	33.3 (7)	71.4 (15)	81.0 (17)	19.0 (4)
*C. perfringens* KT	72.2 (13)	83.3 (15)	100.0 (18)	0 (0)
*C. albicans* ATCC 10239	20.0 (3)	33.3 (5)	60.0 (9)	40.0 (6)

**Table 2:** Prediction by an artificial neural network (FANN) of the inhibition diameter in disk diffusion assays for a specific microorganism (1 output) using the small data input set (*n* = 23). Accuracy is expressed in the percentage of predictions within the given error margin. In parenthesis, total of validating sets correctly predicted within the corresponding error margin.

Strains	Accuracy %			
	ΔID ≤ 5 mm	ΔID ≤ 10 mm	ΔID ≤ 15 mm	ΔID > 15 mm
*S. aureus* ATCC 25923	61.1 (11)	83.3 (15)	100 (18)	0 (0)
*E. coli* ATCC 25922	47.6 (10)	71.4 (15)	81.0 (17)	19.0 (4)
*C. perfringens* KT	38.9 (7)	61.1 (11)	81.0 (17)	19 (4)
*C. albicans* ATCC 10239	26.7 (4)	66.7 (10)	86.7 (13)	13.3 (2)

**Table 3:** Effect of different learning sets on the prediction by an artificial neural network (PredictDemo) of the inhibition diameter in disk diffusion assays for *S*. *aureus* (1 output, small data input set). Accuracy is expressed in the percentage of predictions within the given error margin.

Learning set	Accuracy (%)			
	ΔID ≤ 5 mm	ΔID ≤ 10 mm	ΔID ≤ 15 mm	ΔID > 15 mm
Higher %	54.2	75.0	87.5	8.3
Lower %	41.2	62.5	79.2	20.8
Random	50.0	70.8	87.5	16.7

**Table 4:** Effect of different learning sets on the prediction by an artificial neural network (FANN) of the inhibition diameter in disk diffusion assays for *S*. *aureus* (1 output, small data input set). Accuracy is expressed in the percentage of predictions within the given error margin.

Learning set	Accuracy (%)			
	ΔID ≤ 5 mm	ΔID ≤ 10 mm	ΔID ≤ 15 mm	ΔID > 15 mm
Higher %	50.0	66.7	83.3	16.7
Lower %	37.5	66.7	91.7	8.3
Random	45.8	79.2	91.7	8.3

**Table 5:** Prediction by an artificial neural network (FANN) of the inhibition diameter in disk diffusion assays for a specific microorganism (1 output) using the large data input set. Accuracy is expressed in the percentage of predictions within the given error margin. In parenthesis, total of validating sets correctly predicted within the corresponding error margin.

Strains	Accuracy (%)			
	ΔID ≤ 5 mm	ΔID ≤ 10 mm	ΔID ≤ 15 mm	ΔID > 15 mm
*S*. *aureus* ATCC 25923	66.7 (12)	88.9 (16)	100.0 (18)	0 (0)
*E*. *coli* ATCC 25922	81.0 (17)	85.7 (18)	95.2 (20)	4.8 (1)
*C*. *perfringens* KT	61.1 (11)	72.2 (13)	88.9 (16)	11.1 (2)
*C*. *albicans* ATCC 10239	33.3 (5)	73.3 (11)	86.7 (13)	13.3 (2)

**Table 6:** Simultaneous prediction by an artificial neural network (FANN) of the inhibition diameter in disk diffusion assays for two microorganisms using the large data input set. Accuracy is expressed in the percentage of predictions within the given error margin. In parenthesis, total of inputs correctly predicted within the corresponding error margin.

Strains	Accuracy %			
	ΔID ≤ 5 mm	ΔID ≤ 10 mm	ΔID ≤ 15 mm	ΔID > 15 mm
*S. aureus* ATCC 25923	94.1 (16)	100.0 (17)	100 (17)	0 (0)
*E. coli* ATCC 25922	58.8 (10)	100.0 (17)	100 (17)	0 (0)

**Table 7:** Simultaneous prediction by an artificial neural network (FANN) of the inhibition diameter in disk diffusion assays for three microorganisms using the large data input set. Accuracy is expressed in the percentage of predictions within the given error margin. In parenthesis, total of inputs correctly predicted within the corresponding error margin.

Strains	Accuracy %			
	ΔID ≤ 5 mm	ΔID ≤ 10 mm	ΔID ≤ 15 mm	ΔID > 15 mm
*S*. *aureus* ATCC 25923	83.3 (10)	91.7 (12)	100.0 (12)	0 (0)
*E*. *coli* ATCC 25922	50.0 (6)	91.7 (11)	91.7 (11)	0 (0)
*C*. *perfringens* KT	58.3 (7)	83.3 (10)	91.7 (11)	8.3 (1)
