# Supplementary material for: Application of Artificial Intelligence to the Prediction of the Antimicrobial Activity of Essential Oils
Source: Evid Based Complement Alternat Med. 2015 Sep 17;2015:561024. doi: 10.1155/2015/561024 (PMC4589635; doi:10.1155/2015/561024)
Supplement: Supplementary file 1 — Supplementary Data file contains the composition of the inputs (chemicals), the validation of the ANNs (linear regresions) and the correaltion between real values and predictions. The Excel file contains the raw data with the full composition of the essential oils. [file 561024.f1.pdf]

Supplementary Data

Application of artificial intelligence to the prediction of the antimicrobial activity of essential oils.

Mathieu Daynac, Alvaro Cortes-Cabrera, Jose M. Prieto*.

Submitted to eCAM

TABLE OF CONTENTS

S1: Composition- Antimicrobial activity data availability in the selected 18 articles. In bold, the four micro-organisms that were chosen as output data for the ANN construction. 2

S2: Components of the large input data set.......... 3

S3 different topologies.......... 4

S4: One output experiments, small input data set (n=23), Learning sets, NeuralPredict®.......... 5

S5: One output experiments, small input data set (n=23), Validating sets, NeuralPredict®. 6

S6: One output experiments, small input data set (n=23), predictions, NeuralPredict® 7

S7: One output experiments, small input data set (n=23), Statistics of predictions, NeuralPredict® 8

S8: One output experiments, Statistics of predictions, influence of input selection (Essential oils' components) NeuralPredict® 9

S9: One output experiments, small input data set (n=23), Validating sets, FANN.......... 10

S10: One output experiments, large input data set (75>n>47), Validating sets, FANN. 11

S11: Learning set choice, influence of input selection (Essential oils' components) 12

S12: Two outputs experiments, large input data set (75>n>47), Validating sets, FANN. 13

S13: Two outputs experiments, large input data set (75>n>47), Validating sets, FANN. 14

S1: Composition- Antimicrobial activity data availability in the selected 18 articles. In bold, the four micro-organisms that were chosen as output data for the ANN construction.

	**Micro-organisms**	**No. of data**
Bacteria	*Acinetobacter iwoffii ATCC 19002*	26
	Bacillus cereus ATCC 11778	28
	Clostridium perfringens KUKENS TURKEY	**35**
	Enterobacter aerogenes ATCC 13043	14
	Escherichia coli ATCC 25922	**39**
	Klebsiella pneumoniae ATCC 13883	20
	Moraxella catarrhalis ATCC 49143	14
	Mycobacterium smegmatis CMM 2067	24
	Proteus mirabilis ATCC 7002	15
	Pseudomonas aeruginosa ATCC 27853	32
	Staphylococcus aureus ATCC 25923	**36**
	Streptococcus pneumoniae ATCC 49619	36
Yeast	***Candida albicans ATCC 10239***	**37**
	Candida krusei ATCC 6258	19

S2: Components of the large input data set

limit >10%		limit >5%			
3	Thujene	3	Thujene	90	Thymol
4	α–Pinene	4	α–Pinene	91	Carcavol
6	Camphene	6	Camphene	92	iso-Ascaridole
9	Sabinene	9	Sabinene	101	α – Cubebene
10	β – Pinene	10	β – Pinene	106	Caryophyllene
17	δ– 3 carene	12	α – Myrcene	113	β –Caryophyllene
18	α– Terpinene	17	δ– 3 carene	114	α – Bergamotene
19	p-cymene	18	α– Terpinene	119	cis Muurola-4(14),5 diene
20	Limonene	19	p-cymene	127	Zingiberene
21	1,8 Cineole	20	Limonene	135	γ – Cadinene
23	Eucalyptol	21	1,8 Cineole	147	Nerolidol
26	γ – Terpinene	22	β – Phellandrene	150	Spathulenol
27	cis-Sabinene hydrate	23	Eucalyptol	154	syn-7-Hydroxy-7-anisylnorbornene
34	Linalool	26	γ – Terpinene	158	Cadinol epi-α
39	cis-Menth-2-en-1-ol	27	cis-Sabinene hydrate	160	4a-Methyl-8-methylene-2-naphtale…..
43	trans-Menth-2-en-1-ol	34	Linalool	168	Apiole
45	Menthone	36	Thujone	171	2,2,7,7 Tetramethylcyclo…..
46	Geijerene	39	cis-Menth-2-en-1-ol	172	Cedrenol acetate
47	Camphor	40	Chrysanthenone	175	(E)-B-Santalol acetate
52	Umbellulone	42	trans-Pinocarveol		
53	Borneol	43	trans-Menth-2-en-1-ol		
57	α – Terpineol	45	Menthone		
58	Methyl cavicol	46	Geijerene		
63	γ – Terpineol	47	Camphor		
73	Pulegone	52	Umbellulone		
75	Piperitone	53	Borneol		
78	Piperitone epoxide	55	Terpinen-4-ol		
86	Pregeijerene	57	α – Terpineol		
89	(E) anethole	58	Methyl cavicol		
90	Thymol	61	cis-Dihydrocarveol		
91	Carcavol	63	γ – Terpineol		
92	iso-Ascaridole	70	Bornyl formate		
113	β –Caryophyllene	71	Thymol methyl ether		
147	Nerolidol	73	Pulegone		
154	syn-7-Hydroxy-7-anisylnorbornene	75	Piperitone		
158	Cadinol epi-α	78	Piperitone epoxide		
160	4a-Methyl-8-methylene-2-naphtale…..	85	p-cymen-7-ol		
171	2,2,7,7 Tetramethylcyclo…..	86	Pregeijerene		
175	(E)-B-Santalol acetate	89	(E) anethole		

S3 DIFFERENT TOPOLOGIES.

	Topo 1	Topo 2	Topo 3	Topo 4	Topo 5
Hidden layers	30 → 15 → 5	20 → 30 → 5	10 → 20 → 30	20 → 20 → 20	40 → 30 → 20 → 10
Network overview	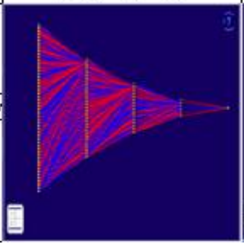	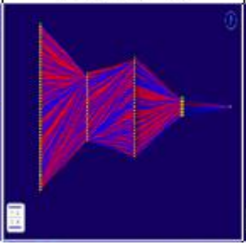	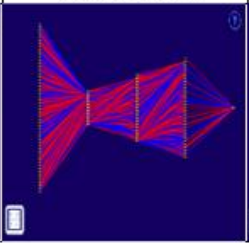	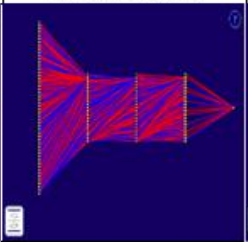	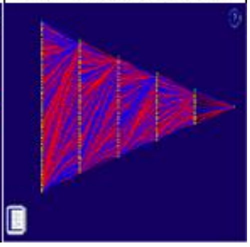

In red, neuronal connections with higher contribution to the output.

S4: ONE OUTPUT EXPERIMENTS, SMALL INPUT DATA SET (N=23), LEARNING SETS, NEURALPREDICT®.

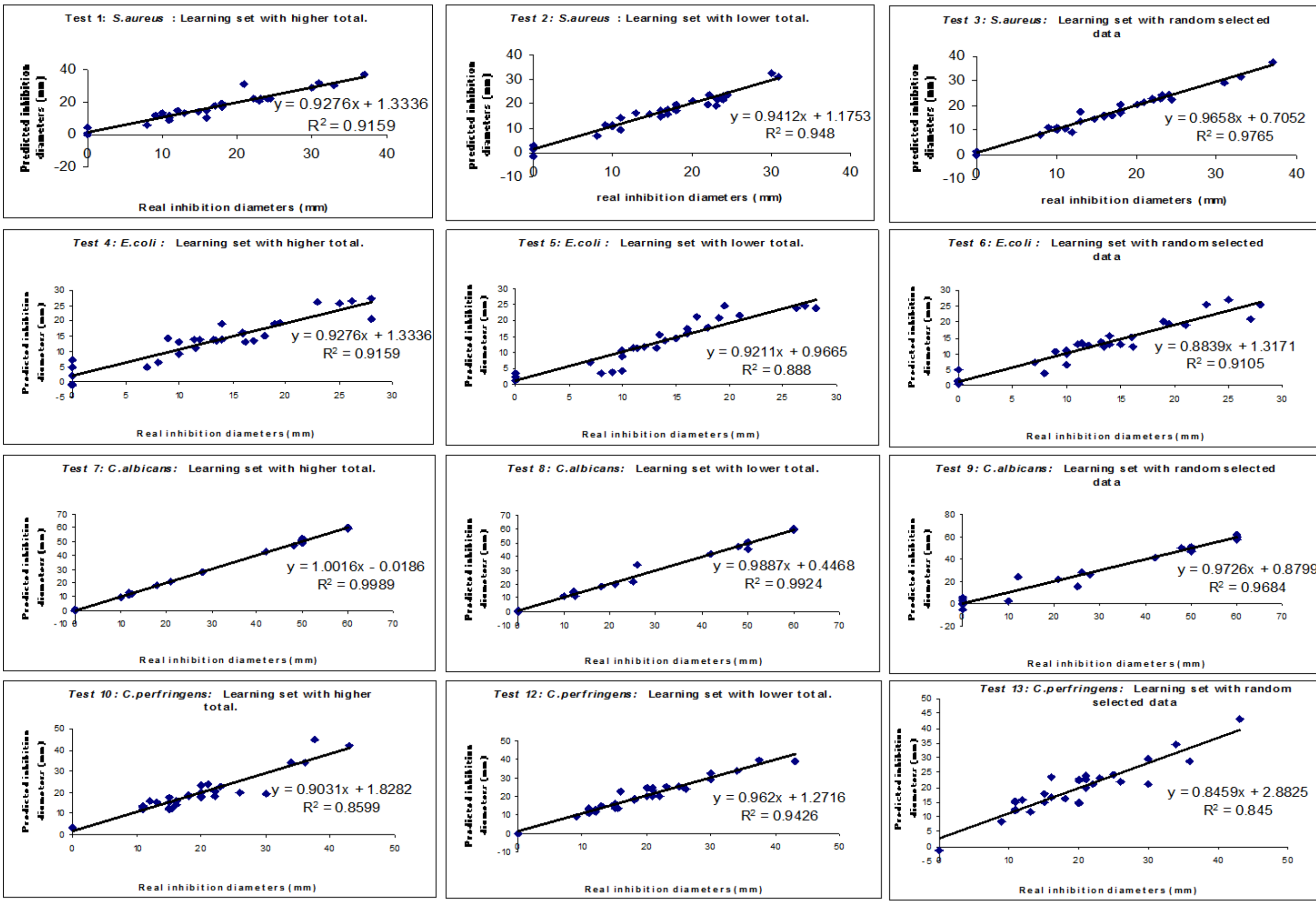

S5: ONE OUTPUT EXPERIMENTS, SMALL INPUT DATA SET (N=23), VALIDATING SETS, NEURALPREDICT®.

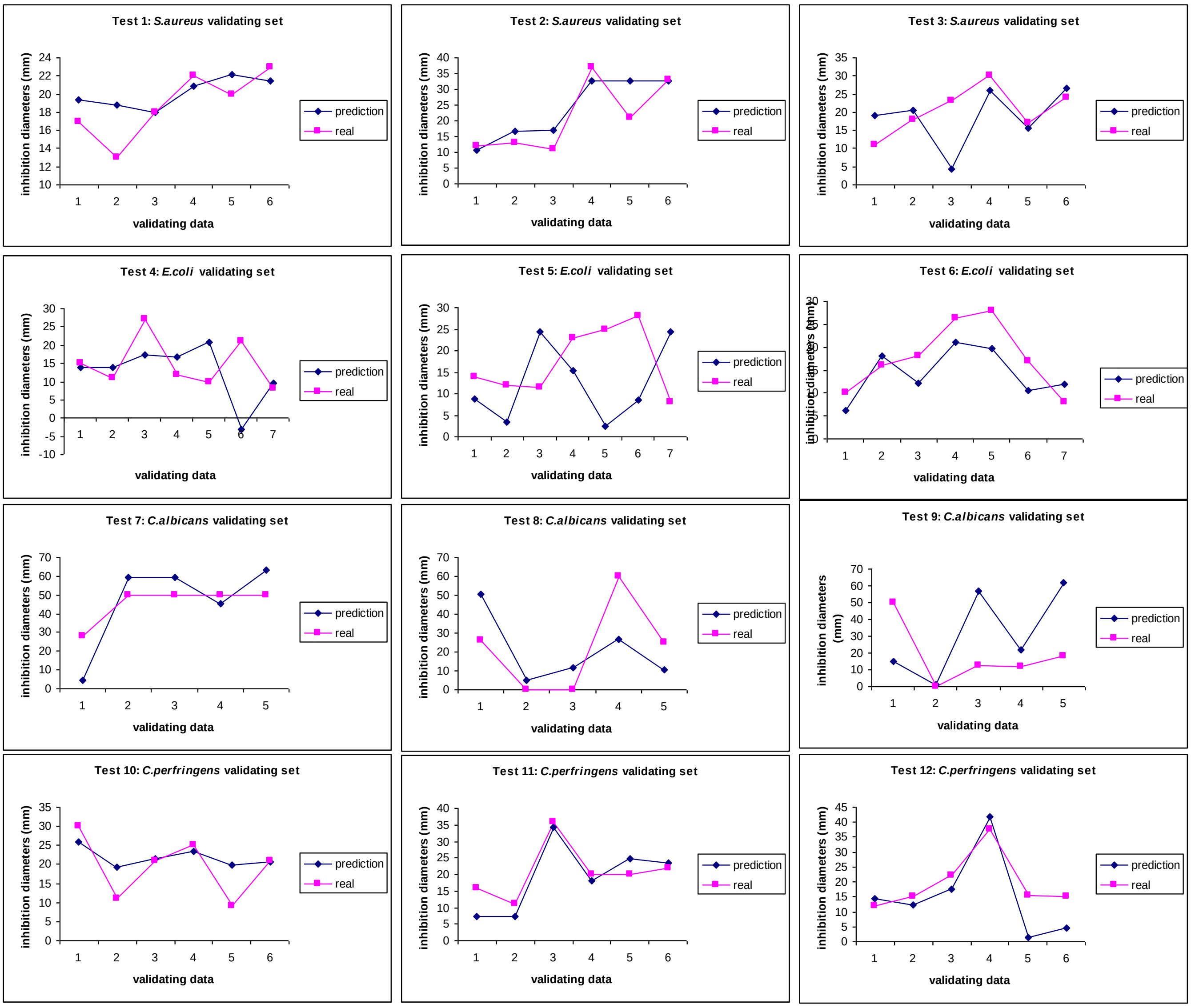

S6: One output experiments, small input data set (n=23), predictions, NeuralPredict® .

Table ?	Validating sets										
Prediction No	Test	No of sample	ID (mm)	ID calc(mm)	Δ ID (mm)	Prediction No	Test	No of sample	ID (mm)	ID calc(mm)	Δ ID (mm)
1	TEST 1 *S.aureus*	1	17	19.4	2.4	40	TEST 7 *C.albicans*	1	28	4.6	23.4
2		2	13	18.8	5.8	41		2	50	59.3	9.3
3		3	18	18.0	0.0	42		3	50	59.4	9.4
4		4	22	20.9	1.1	43		4	50	45.6	4.4
5		5	20	22.2	2.2	44		5	50	63.2	13.2
6		6	23	21.5	1.5	45	TEST 8 *C.albicans*	1	26	50.8	24.8
7	TEST 2 *S.aureus*	1	12	10.8	1.2	46		2	0	4.8	4.8
8		2	13	16.8	3.8	47		3	0	11.5	11.5
9		3	11	16.9	5.9	48		4	60	26.7	33.3
10		4	37	32.7	4.3	49		5	25	10.4	14.6
11		5	21	32.7	11.7	50	TEST 9 *C.albicans*	1	50	15.2	34.8
12		6	33	32.7	0.3	51		2	0	1.0	1.0
13	TEST 3 *S.aureus*	1	11	19.2	8.2	52		3	12.5	56.8	44.3
14		2	18	20.6	2.6	53		4	12	22.2	10.2
15		3	23	4.3	18.7	54		5	18	61.7	43.7
16		4	30	26.0	4.0	55	TEST 10 *C.perfringens*	1	30	25.9	4.1
17		5	17	15.7	1.3	56		2	11	19.2	8.2
18		6	24	26.6	2.6	57		3	21	21.4	0.4
19	TEST 4 *E.coli*	1	15	13.8	1.2	58		4	25	23.4	1.6
20		2	11	13.8	2.8	59		5	9	19.7	10.7
21		3	27	17.2	9.8	60		6	21	20.6	0.4
22		4	12	16.7	4.7	61	TEST 11 *C.perfringens*	1	16	7.2	8.8
23		5	10	20.9	10.9	62		2	11	7.2	3.8
24		6	21	-3.1	24.1	63		3	36	34.3	1.7
25		7	8	9.5	1.5	64		4	20	18.0	2.0
26	TEST 5 *E.coli*	1	14	8.8	5.2	65		5	20	24.7	4.7
27		2	12	3.4	8.6	66		6	22	23.6	1.6
28		3	11.5	24.5	13.0	67	TEST 12 *C.perfringens*	1	12	14.3	2.3
29		4	23	15.3	7.7	68		2	15	12.3	2.7
30		5	25	2.4	22.6	69		3	22	17.5	4.5
31		6	28	8.5	19.5	70		4	37.5	41.8	4.3
32		7	8	24.4	16.4	71		5	15.5	1.6	13.9
33	TEST 6 *E.coli*	1	10	6.1	3.9	72		6	15	4.4	10.6
34		2	16	18.0	2.0						
35		3	18	12.2	5.8			Number	%		
36		4	26.25	21.1	5.2		ΔID< 5mm	36	50.0		
37		5	28	19.7	8.3		ΔID< 10mm	51	70.8		
38		6	17	10.5	6.5		ΔID< 15mm	61	84.7		
39		7	8	11.8	3.8		ΔID> 15mm	11	15.3		

S7: One output experiments, small input data set (n=23), Statistics of predictions, NeuralPredict® .

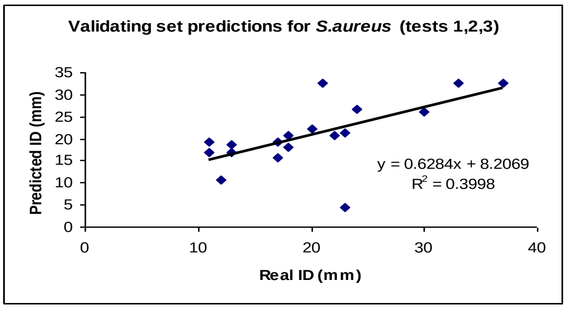

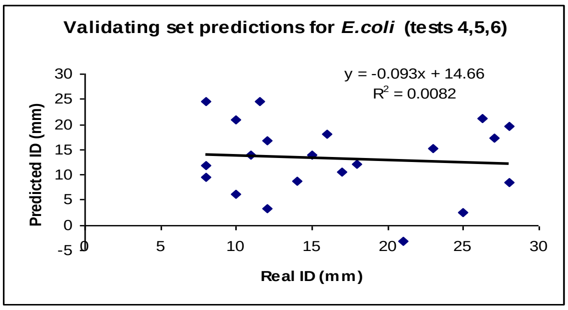

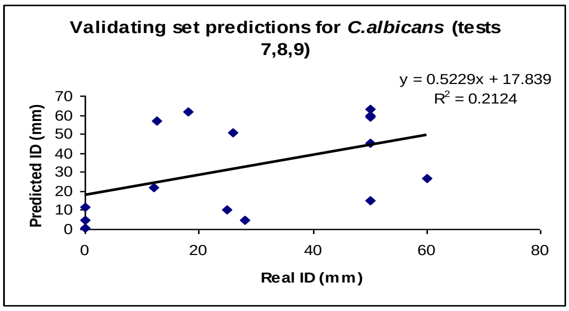

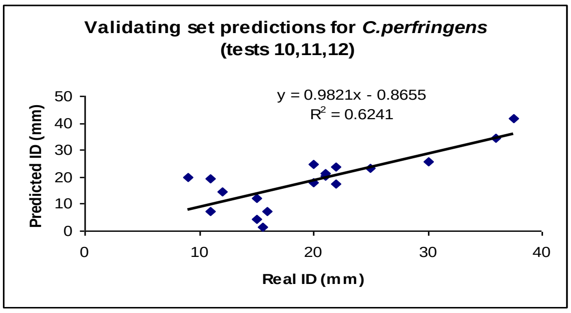

S.aureus	Number	%	*E.coli*	Number	%
ΔID ≤ 5mm	13	72.2	ΔID ≤ 5mm	7	33.3
ΔID ≤ 10mm	16	88.9	ΔID ≤ 10mm	15	71.4
ΔID ≤15mm	17	94.4	ΔID ≤ 15mm	17	81.0
ΔID> 15mm	1	5.6	ΔID> 15mm	4	19.0
C.albicans	Number	%	*C.perfringens*	Number	%
ΔID ≤ 5mm	3	20.0	ΔID ≤ 5mm	13	72.2
ΔID ≤ 10mm	5	33.3	ΔID ≤ 10mm	15	83.3
ΔID ≤ 15mm	9	60.0	ΔID ≤ 15mm	18	100.0
ΔID> 15mm	6	40.0	ΔID> 15mm	0	0.0

S8: One output experiments, Statistics of predictions, influence of input selection (Essential oils' components) NeuralPredict® .

	ΔID< 5mm %	ΔID< 10mm %	ΔID< 15mm %	ΔID> 15mm %
learning set with higher total (tests 1,4,7,10)	54.2	75.0	87.5	8.3
learning set with lower total (tests 2,5,8,11)	41.2	62.5	79.2	20.8
learning set with random selection (tests 3,6,9,12)	50.0	70.8	87.5	16.7

S9: One output experiments, small input data set (n=23), Validating sets, FANN.

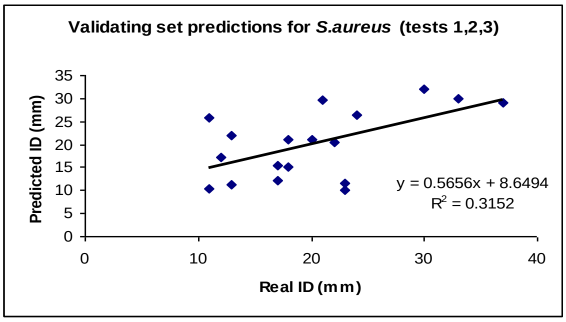

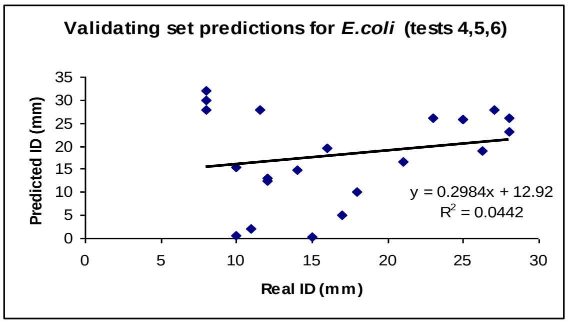

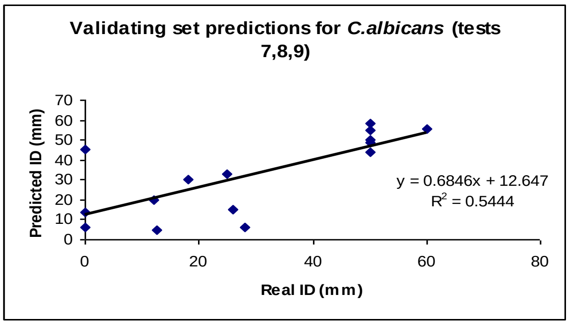

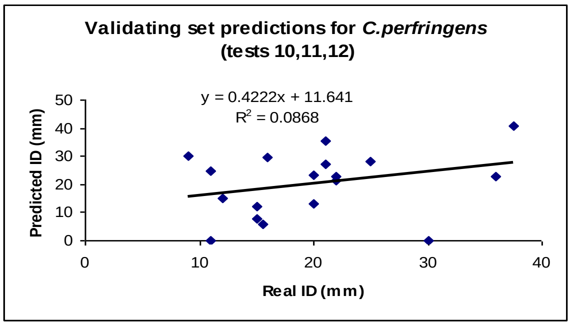

S.aureus	**Number**	**%**	***E.coli***	**Number**	**%**
**ΔID ≤ 5mm**	**11**	**61.1**	**ΔID ≤ 5mm**	**10**	**47.6**
**ΔID ≤ 10mm**	**15**	**83.3**	**ΔID ≤ 10mm**	**15**	**71.4**
**ΔID ≤15mm**	**18**	**100.0**	**ΔID ≤ 15mm**	**17**	**81.0**
**ΔID> 15mm**	**0**	**0.0**	**ΔID> 15mm**	**4**	**19.0**
C.albicans	**Number**	**%**	***C.perfringens***	**Number**	**%**
**ΔID ≤ 5mm**	**4**	**26.7**	**ΔID ≤ 5mm**	**7**	**38.9**
**ΔID ≤ 10mm**	**10**	**66.7**	**ΔID ≤ 10mm**	**11**	**61.1**
**ΔID ≤ 15mm**	**13**	**86.7**	**ΔID ≤ 15mm**	**16**	**88.9**
**ΔID> 15mm**	**2**	**13.3**	**ΔID> 15mm**	**2**	**11.1**

S10: One output experiments, large input data set (75>n>47), Validating sets, FANN.

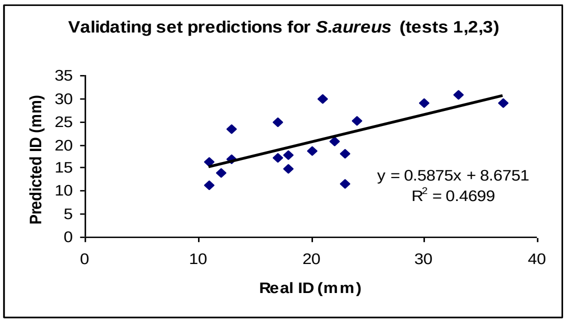

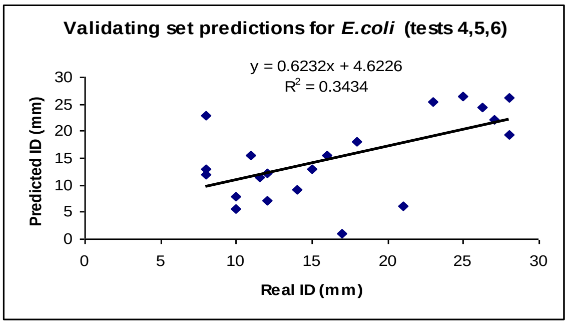

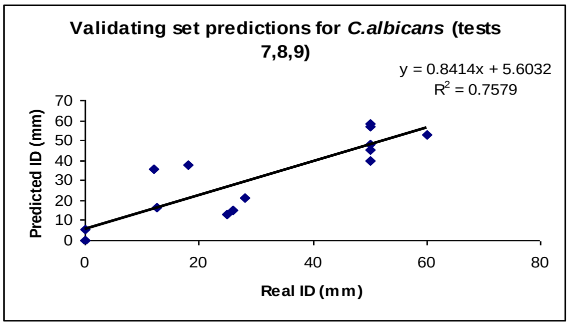

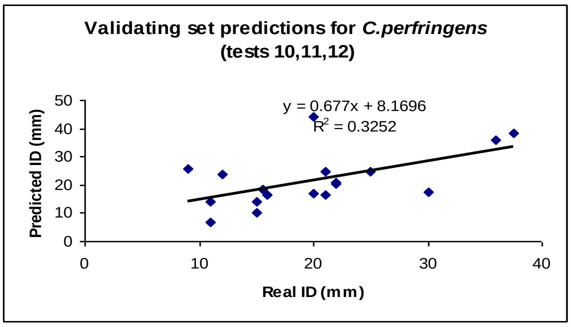

S.aureus	Number	%	*E.coli*	Number	%
ΔID ≤ 5mm	12	66.7	ΔID ≤ 5mm	17	81.0
ΔID ≤ 10mm	16	88.9	ΔID ≤ 10mm	18	85.7
ΔID ≤15mm	18	100.0	ΔID ≤ 15mm	20	95.2
ΔID> 15mm	0	0.0	ΔID> 15mm	1	4.8
C.albicans	Number	%	*C.perfringens*	Number	%
ΔID ≤ 5mm	5	33.3	ΔID ≤ 5mm	11	61.1
ΔID ≤ 10mm	11	73.3	ΔID ≤ 10mm	13	72.2
ΔID ≤ 15mm	13	86.7	ΔID ≤ 15mm	16	88.9
ΔID> 15mm	2	13.3	ΔID> 15mm	2	11.1

S11: Learning set choice, influence of input selection (Essential oils' components)

	ΔID< 5mm %	ΔID< 10mm %	ΔID< 15mm %	ΔID> 15mm %
**learning set with higher total (tests 1,4,7,10)**	50.0	66.7	83.3	16.7
**learning set with lower total (tests 2,5,8,11)**	37.5	66.7	91.7	8.3
**learning set with random selection (tests 3,6,9,12)**	45.8	79.2	91.7	8.3

S12: Two outputs experiments, large input data set (75>n>47), Validating sets, FANN.

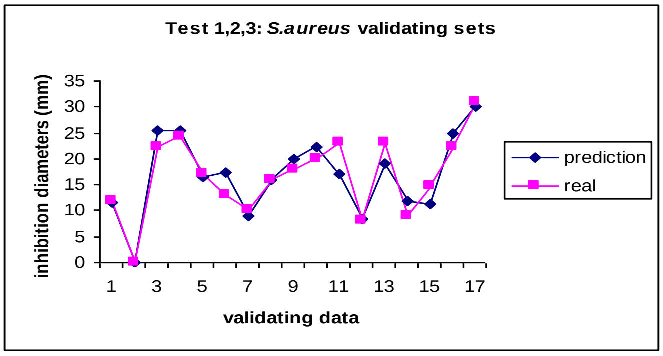

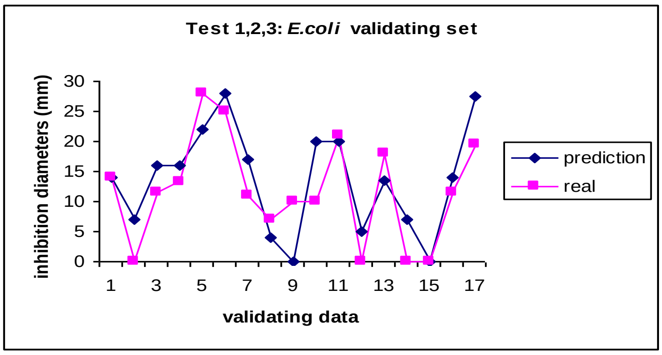

S.aureus	Number	%	*E.coli*	Number	%
ΔID ≤ 5mm	16	94,1	ΔID ≤ 5mm	10	58,8
ΔID ≤ 10mm	17	100,0	ΔID ≤ 10mm	17	100,0
ΔID ≤ 15mm	17	100,0	ΔID ≤ 15mm	17	100,0
ΔID > 15mm	0	0,0	ΔID > 15mm	0	0,0

S13: Two outputs experiments, large input data set (75>n>47), Validating sets, FANN.

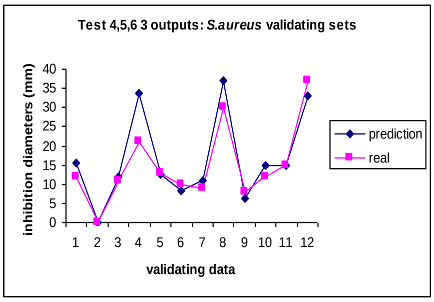

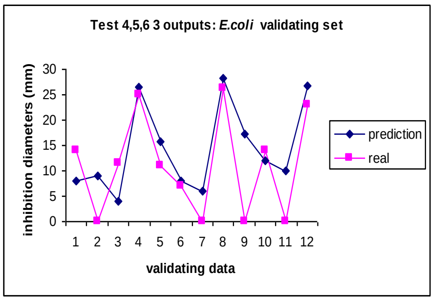

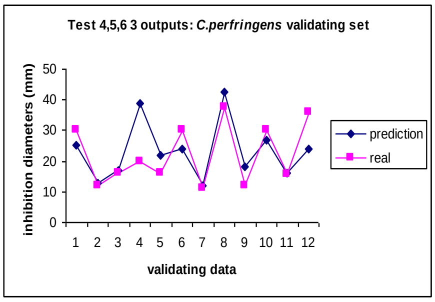

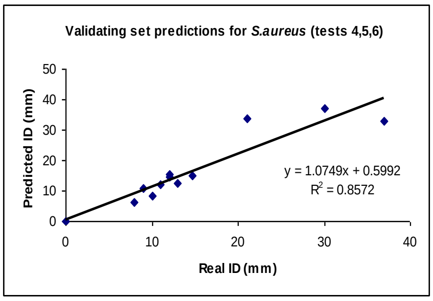

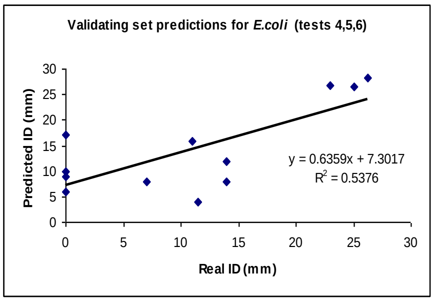

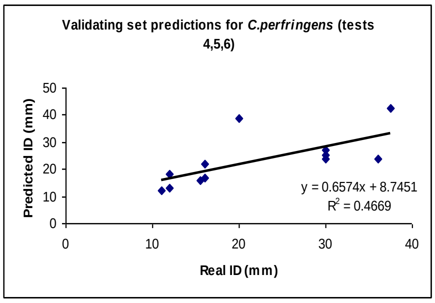

S.aureus	Number	%	*E.coli*	Number	%	*C.perfringens*	Number	%
ΔID ≤ 5mm	10	83,3	ΔID ≤ 5mm	6	50,0	ΔID ≤ 5mm	7	58,3
ΔID ≤ 10mm	11	91,7	ΔID ≤ 10mm	11	91,7	ΔID ≤ 10mm	10	83,3
ΔID ≤ 15mm	12	100,0	ΔID ≤ 15mm	11	91,7	ΔID ≤ 15mm	11	91,7
ΔID > 15mm	0	0,0	ΔID > 15mm	1	8,3	ΔID > 15mm	1	8,3
